# High-Resolution Ex Vivo Confocal Microscopy to Distinguish Cutaneous T-Cell Lymphoma from Inflammatory Mimickers

**DOI:** 10.3390/bioengineering13101114

**Published:** 2026-09-24

**Authors:** Daniela Hartmann, Aimée Braun, Benjamin Kendziora, Katharina Wex, Paulina Pabst, Andreas Ohlmann, Elke C. Sattler, Maximilian Deußing

**Affiliations:** 1 Department of Dermatology and Allergy, LMU Medizin, LMU University Hospital, Ludwig-Maximilians-Universität Munich, 80337 Munich, Germany; 2 Department of Dermatology and Allergy, Munich Municipal Hospital, 80337 Munich, Germany; 3 Department of Ophthalmology, LMU University Hospital, LMU Munich, 80336 Munich, Germany

**Keywords:** dermatology, oncology, non-invasive imaging, digital pathology, bedside histology

## Abstract

Cutaneous T-cell lymphoma (CTCL) often clinically resembles inflammatory dermatoses such as eczema, making accurate and timely diagnosis challenging. Since early differentiation is critical for appropriate treatment and prognosis, fast and reliable diagnostic tools are needed. Ex vivo confocal laser scanning microscopy (EVCM) has emerged as a promising technique for rapid, high-resolution tissue assessment. As CTCL is difficult to identify based on clinical presentation alone and is frequently misdiagnosed as eczema, this study assessed the diagnostic accuracy and clinical utility of EVCM in differentiating CTCL from eczema, as well as interobserver agreement among examiners with varying experience. Fifty specimens (25 eczema, 25 CTCL) were imaged using EVCM (Vivascope 2500 M-G4; Vivascope GmbH, Munich, Germany). Three independent, blinded dermatologists with different experience levels classified lesions based solely on digital H&E-like images. Sensitivity, specificity, and predictive values were calculated. EVCM showed high diagnostic accuracy, with a mean concordance rate of 82.67%. The dermatohistopathologist achieved the highest accuracy (84%), followed by two trained dermatologists (82% each). Sensitivity ranged from 0.80 to 0.88 and specificity from 0.76 to 0.84, with comparable predictive values and almost perfect interobserver agreement (Fleiss’ κ = 0.89). Misclassifications mainly involved the same diagnostically challenging cases across both entities. EVCM demonstrated high diagnostic accuracy across examiners of varying experience, supporting its potential as a rapid bedside tool for distinguishing CTCL from eczema. While not replacing histopathology as the gold standard, it may meaningfully complement routine diagnostics. Integration of immunohistochemistry or artificial intelligence could further enhance diagnostic precision.

## 1. Introduction

Cutaneous lymphomas are broadly classified into two major groups: cutaneous B-cell lymphomas (CBCLs) and cutaneous T-cell lymphomas (CTCLs). The latter predominates and accounts for approximately 75–80% of the cases. CTCLs represent a heterogeneous group of extranodal non-Hodgkin lymphomas, which includes mycosis fungoides (MF), Sézary syndrome (SS), CD30-positive lymphoproliferative disorders, as well as rarer aggressive subtypes such as subcutaneous panniculitis-like T-cell lymphoma and primary cutaneous γ/δ T-cell lymphoma, which show distinct incidences worldwide [1]. MF represents the most frequent subtype, accounting for approximately 62% of all CTCL cases, while the distribution of rarer variants varies with ethnicity [2]. The global incidence of CTCL lies at approximately 6.4 cases per million individuals. Epidemiological studies demonstrate a male predominance of cutaneous T-cell lymphoma [2,3]. Risk increases with age, with a median age of approximately 54 years at the time of diagnosis, although rare cases occur in children and adolescents also [4]. CTCL is rarely diagnosed during its early stages, leaving many cases undetected, which is reflected in an underestimation of its true incidence [5] and hence adversely affects patient survival [6,7].

The diagnosis of CTCL is often delayed due to the slow progression of the disease and its initial presentation with nonspecific skin lesions that can resemble common inflammatory dermatoses such as eczema or psoriasis, frequently lacking the classical hallmarks of CTCL. Histopathologically, CTCL can mimic a wide range of common and rare inflammatory skin conditions, further contributing to a delay in accurate diagnosis [8,9]. As a result, patients are often misdiagnosed, leading to ineffective therapies, prolonged suffering, and increased morbidity and mortality [10,11]. While corticosteroid treatment can provide temporary relief from symptoms, certain immunosuppressive therapies, such as tumor necrosis factor α (TNF α) inhibitors used in psoriasis, can potentially accelerate disease progression [8]. Beyond its physical manifestations, the psychological burden and social impact of CTCL significantly lowers patient’s quality of life [10,11,12].

Currently, histopathological examination represents the gold standard for diagnostic assessment in dermatology. By utilizing multiple section levels, various staining protocols, and specific immunohistochemical analyses, this approach offers a detailed and highly specific diagnostic tool that frequently provides clarity in otherwise inconclusive cases. Despite its central role in modern diagnostics, the major limitation of histopathology lies in its time requirements—processes such as fixation, staining, and section preparation may often take several days to weeks to complete.

Ex vivo confocal laser scanning microscopy (EVCM) has shown promising results as a rapid and reliable diagnostic tool for the real-time evaluation of freshly excised tissue. It provides cellular and architectural information at approximately histopathological resolution and may enable morphological assessment before conventional histopathological tissue processing is completed. Its potential has been demonstrated in various benign and malignant skin tumors, including squamous cell carcinoma (SCC) [13,14], malignant melanoma (MM) [15], and basal cell carcinoma (BCC), as well as in inflammatory skin diseases such as lichen planus (LP) [16], eczematous lesions, and psoriasis vulgaris [17]. These advantages may facilitate a more rapid diagnostic assessment and could potentially support the early identification of CTCL. Based on these findings, the current study aims to evaluate the potential application of EVCM in CTCL.

The primary objective of this study was to establish the extent to which CTCL could be identified based on EVCM images of biopsy specimens. Three independent examiners with dermatohistopathological expertise were requested to distinguish between 50 cases, including 25 eczema and 25 CTCL samples. In a second step, misclassified cases were analyzed to identify potential sources of diagnostic error and improve the evaluation process.

## 2. Materials and Methods

To ensure appropriate patient selection, the study cohort included 25 cases of CTCL and 25 cases of eczema in a time period between October 2020 and March 2025 at the Department of Dermatology and Allergy, University Hospital, Ludwig Maximilian University Munich (LMU). Of the 25 CTCL cases, 18 (72%) were histopathologically classified as mycosis fungoides (MF), while the remaining 7 (28%) were classified as peripheral T-cell lymphoma without further subtyping (PTCL-NOS). In all cases, both CTCL and eczema had been considered as potential diagnoses based on the clinical morphology of the lesions. This selection strategy allowed the inclusion of patients in whom a definitive clinical distinction between the two entities was challenging or not achievable. As a consequence, a more extended diagnostic workup was needed, allowing the evaluation of the usefulness and accuracy of EVCM as a bedside imaging tool in routine clinical practice. Patients of all ages with Fitzpatrick skin types I to IV were included in the study, including both individuals who had not yet been diagnosed as well as those who had been diagnosed but were currently on a treatment break, uncertain whether new lesions represented recurrences of CTCL or new eczematous lesions. Written informed consent was obtained before admission from all participants, in accordance with the approval of the local ethics committee of LMU University Hospital (Ref. No. 19-150 and Ref. No. 23-0393).

As part of the diagnostic biopsy procedure, skin samples were collected and immediately covered in a saline solution to allow rapid EVCM imaging. Following a standardized protocol, each tissue sample was immersed in sequence for 30 s in acridine orange (AO; 0.04 mmol/L; Sigma-Aldrich, St. Louis, MO, USA), phosphate-buffered saline (Dulbecco’s PBS; pH 7.49) and physiologic saline solution (NaCl, 9 g/L). The stained tissue samples were then positioned with the freshly cut surface facing the laser scanner, between two magnetic glass slides secured with specialized sponges, to achieve optimal visualization of the epidermis and dermis. Each sample was scanned under identical parameters to allow reliable comparison and differentiation.

Next, EVCM imaging was performed using the VivaScope 2500 M-G4 (VivaScope, Munich, Germany), which simultaneously uses two lasers of different wavelengths, 488 nm (blue, fluorescence) and 638 nm (red, reflection), to generate digitally stained H&E-like images. The system provides a horizontal optical resolution of <1.25 μm and a vertical optical resolution of <5.0 μm, with an imaging depth of up to 200 μm. Images are acquired at a resolution of 1024 × 1024 pixels, corresponding to 0.5 μm/pixel, with a magnification of up to 550×. The system is equipped with a 38× water-immersion objective with a numerical aperture of 0.85 [18].

After imaging, the entire fresh tissue samples were fixed in formalin and subjected to the independent, standardized routine dermatohistopathological workup at the in-house histopathology unit of the Department of Dermatology and Allergy, University Hospital, LMU Munich. All skin samples underwent conventional histopathological examination, which served as the diagnostic gold standard. Evaluations were carried out by experienced dermatohistopathologists following standardized protocols, with immunostaining applied where appropriate. These findings were used to validate the diagnoses, leading to 50 suitable cases with their respective diagnoses.

The 50 EVCM-acquired images were incorporated into a randomized and anonymized quiz containing both an overview of the entire sample and a close-up of the epidermis and dermis. Three blinded, independent experts—an EVCM-trained dermatohistopathologist with extensive histopathological expertise (Examiner A; D.H.), an EVCM-unexperienced but dermatohistopathology-experienced investigator (Examiner B; B.K.), and an EVCM-trained dermatologist without dermatohistopathology experience (Examiner C; M.D.)—were assigned the task of differentiating between eczematous lesions and cutaneous T-cell lymphoma. Each examiner could only base their decision solely on the EVCM image without any further information concerning the patient or their medical history, nor the evaluations conducted by other examiners.

In identifying eczema samples (Figure 1), examiners focused on epidermal changes including spongiosis, acanthosis, and exocytosis of inflammatory cells, as well as dermal changes, including perivascular lymphocytic infiltrates in the superficial dermis and the presence of edema. By contrast, cutaneous T-cell lymphoma is characterized by features such as epidermotropism and, in some cases, Pautrier microabscesses, defined as clusters of atypical lymphocytes within the epidermis. However, these are not universally present and thereby sometimes not detectable. The examination of the dermis in CTCL reveals a superficial band-like infiltrate of atypical T-cells in the papillary dermis and disruption of normal dermal architecture [19].

Once all three examiners had completed their assessments independently, without knowing the results from the classical histopathological path, the descriptive statistical analysis was performed using Microsoft Excel for Microsoft 365 MSO (Version 2508 Build 16.0.19127.20192). Diagnostic performance (sensitivity, specificity, positive and negative predictive value, and overall accuracy) was calculated for each examiner, with 95% confidence intervals derived using the Wilson score method, which provides better coverage than the normal approximation for proportions in small samples [20]. Interobserver agreement among the three examiners was quantified using Fleiss’ κ [21], with the large-sample standard error and 95% confidence interval calculated according to Fleiss, Levin and Paik [22]; κ values were interpreted according to the criteria of Landis and Koch [23]. Differences in diagnostic accuracy between examiners were assessed using Cochran’s Q test [24] across all three raters, followed by pairwise exact McNemar tests [25] as post hoc comparisons. Misclassification rates between CTCL subtypes were compared using Fisher’s exact test, given the small subgroup sizes [26]. These additional analyses were performed using IBM SPSS Statistics for Windows, Version 32.0 (IBM Corp., Armonk, NY, USA). A *p*-value < 0.05 was considered statistically significant.

## 3. Results

A total of 50 EVCM images were analyzed, including 25 cases of eczema and 25 cases of CTCL. The cohort comprised 15 female (30%) and 35 male (70%) patients. The median age of the subjects was 62.3 years, with median ages of 60.6 and 64.2 years among patients with eczema and CTCL, respectively.

As depicted in Figure 2, EVCM demonstrated a high diagnostic accuracy in distinguishing CTCL from eczema, with an average concordance of 83% among all three investigators. The EVCM-trained dermatohistopathologist (Examiner A, D.H.) achieved the highest accuracy with 42/50 cases correctly diagnosed, or 84%, closely followed by the two EVCM-trained dermatologists with dermatohistopathology experience, specifically Examiner B (B.K.) and Examiner C (M.D.), at 41/50 and 82%.

The diagnostic performance of each examiner in differentiating between CTCL and eczema is summarized in Table 1. The senior EVCM specialist with extensive histopathological expertise, Examiner A, reached a sensitivity of 0.84 (95% CI: 0.65–0.94), a specificity of 0.84 (95% CI: 0.65–0.94), and positive as well as negative predictive values (PPV and NPV) of 0.84 (95% CI: 0.65–0.94 for both). Examiner B reached a sensitivity of 0.80 (95% CI: 0.61–0.91), a specificity of 0.84 (95% CI: 0.65–0.94), a PPV of 0.83 (95% CI: 0.64–0.93), and an NPV of 0.81 (95% CI: 0.62–0.91), while Examiner C demonstrated a sensitivity of 0.88 (95% CI: 0.70–0.96), a specificity of 0.76 (95% CI: 0.57–0.89), a PPV of 0.79 (95% CI: 0.60–0.90), and an NPV of 0.86 (95% CI: 0.67–0.95). Overall accuracy was 0.84 (95% CI: 0.71–0.92) for Examiner A, and 0.82 (95% CI: 0.69–0.90) for both Examiners B and C.

Analysis of the misclassified EVCM images revealed that, of the 8 and 9 misclassified EVCM cases, 7 were identical, comprising 4 eczema and 3 CTCL cases. One further CTCL case was misclassified by Examiners A and B but correctly identified by Examiner C. The remaining 2 incorrectly classified pictures, one eczema and one CTCL, were independently misclassified by Examiners B and C. Among the CTCL cases, misclassification (by at least one examiner) occurred in 4 of 18 (22.2%) mycosis fungoides cases and 1 of 7 (14.3%) PTCL-NOS cases; this difference was not statistically significant (Fisher’s exact test, *p* = 1.00).

Interobserver agreement among the three examiners was almost perfect, with a Fleiss’ κ of 0.89 (95% CI: 0.73–1.00), consistent with the finding that only 4 of the 50 lesions were classified inconsistently between examiners. Cochran’s Q test showed no significant difference in overall diagnostic accuracy among the three examiners (Q = 0.50, df = 2, *p* = 0.78). Pairwise exact McNemar tests likewise revealed no significant differences in accuracy between any two examiners (A vs. B, A vs. C, B vs. C: all *p* = 1.00), indicating that the numerically lower specificity and PPV observed for Examiner C did not reach statistical significance and may reflect sampling variability rather than a systematic effect of examiner experience.

Furthermore, a focused re-analysis of the misclassified images identified three major sources of diagnostic challenge. As shown in Figure 3, most cases were affected by these limitations and therefore misdiagnosed.

The first concerns the lesions’ inflammatory component. The infiltration of heterogeneous inflammatory cells may lead to crowding and cellular overlap. Given that EVCM acquires only a single optical section per acquisition, unlike the multi-level tissue analysis made possible in histopathology, this may hinder a reliable single-cell evaluation and result in a sampling error. Consequently, atypical lymphocytes characteristic of CTCL may thus be hidden or overlooked when distinct lymphocyte subsets cannot be adequately resolved.

The second limitation concerns specimen staining. Since staining remains a manual laboratory procedure, variability in stain uptake is common: some of the tissue samples may be overstained, while others may fail to incorporate sufficient contrast; hence, resulting in EVCM images that are excessively hyper- or hypointense. The challenging imaging conditions adversely affect the evaluation of tissue architecture and cytomorphology, which are critical for accurate differential diagnosis, thereby increasing the likelihood of misclassification.

The third limitation entails specimen fixation and mounting. Accurate architectural assessment requires proper positioning of the epidermis on the glass slide, but mechanical distortion during biopsy acquisition or improper handling can disrupt epidermal morphology. The loss of these key diagnostic features may contribute to misinterpretation. Interestingly, several of the misclassified specimens required additional immunohistochemical analyses, since conventional staining alone was insufficient to establish a definitive diagnosis. Such findings underscore that even histopathology—the current gold standard—relies on extended diagnostic evaluation in cases with ambiguous or challenging morphological presentation.

This further highlights a current limitation of the EVCM technique. Artificial HE-like images can be generated using existing staining protocols and different wavelength settings, thus enabling tissue assessment similar to conventional histopathology. However, the method remains limited in diagnostically challenging cases or specific entities such as CTCL. In these cases, immunohistochemistry (IHC) provides important diagnostic support. IHC uses antibodies to detect T-cell-specific markers in skin biopsies. Enzyme-conjugated secondary antibodies react with chromogens like DAB and yield a visible signal, thus highlighting malignant T-cells. Currently, EVCM does not provide the ability for combination with IHC. Therefore, evaluation remains restricted to HE-based analysis alone.

## 4. Discussion

Our data indicate that EVCM allows consistently high diagnostic accuracy among examiners, with the most balanced performance observed in the highly experienced EVCM specialist. In contrast, dermatohistopathologists without dedicated EVCM expertise tend to emphasize sensitivity at the expense of specificity, while the performance of individual examiners with EVCM training varied slightly. However, these descriptive differences were not statistically significant (Cochran’s Q test, *p* = 0.78; pairwise exact McNemar tests, all *p* = 1.00), and interobserver agreement across the three examiners was almost perfect (Fleiss’ κ = 0.89, 95% CI: 0.73–1.00), indicating that EVCM assessment is highly reproducible even across examiners with differing levels of experience.

These findings indicate that EVCM provides high diagnostic accuracy in distinguishing CTCL from eczema across different examiners. The EVCM specialist achieved the most balanced performance. While descriptive trends suggested some influence of examiner background and expertise, this was not confirmed statistically, and EVCM offers robust and clinically valuable results. More frequent use of EVCM in routine biopsy evaluation could enhance examiner experience and further improve diagnostic accuracy in distinguishing skin lesions with overlapping clinical features.

Misclassification frequency did not differ significantly between mycosis fungoides and PTCL-NOS cases (4/18, 22.2% vs. 1/7, 14.3%; Fisher’s exact test, *p* = 1.00), although this comparison was limited by the small number of PTCL-NOS cases. As rarer CTCL subtypes such as Sézary syndrome and CD30-positive lymphoproliferative disorders were not represented, or only minimally represented, in our cohort, our findings should be considered most directly applicable to mycosis fungoides and PTCL-NOS rather than to CTCL as a biologically heterogeneous entity as a whole.

The aim of our study was to evaluate the potential role of EVCM as a diagnostic tool in daily clinical practice, especially underlining the advantage of providing rapid bedside assessments. In contrast, dermatohistopathology, the current gold standard, requires multistep procedures, which may take several hours to days to complete. A preliminary bedside evaluation using EVCM could therefore provide timely diagnostic support. However, it is important to note that EVCM is not intended to replace histopathology, but rather to complement it, integrating technological innovation into diagnostic progress.

Several studies investigating the use of the EVCM in common skin diseases, such as squamous cell carcinoma (SCC) [13], malignant melanoma (MM) [15], lichen planus (LP) [16] and eczematous lesions [17], have documented its promising diagnostic potential. In contrast, research on cutaneous lymphomas has predominantly focused on epidemiology, pathogenesis, and therapeutic strategies. To date, CTCL has not been specifically addressed in the context of EVCM imaging, apart from a single study on rare skin diseases, which included one case of CTCL to explore its potential diagnostic applicability [27].

However, the relatively small sample size of 25 eczema and CTCL cases each, combined with the limited number of examiners, constitutes an important limitation of this study. Given this sample size, the statistical comparisons between examiners (Cochran’s Q and McNemar tests) may have been underpowered to detect smaller, potentially clinically relevant differences related to examiner experience; the absence of statistical significance should therefore not be over-interpreted as definitive proof of full equivalence between examiners. Similarly, the comparison between mycosis fungoides and PTCL-NOS cases was based on a small number of PTCL-NOS cases (*n* = 7) and should be interpreted with caution. Within the mycosis fungoides subgroup, disease stage (patch, plaque, or tumor stage) was not systematically available for all cases, precluding a dedicated analysis of diagnostic performance in early patch-stage disease, which likely represents the most diagnostically challenging scenario for differentiating CTCL from eczema; this should be addressed in future prospective studies with systematic stage documentation.

Given that CTCL is a rare disease in Germany, with an estimated incidence of 3.65 to 3.92 per 100,000 inhabitants in 2017 [28], the recruitment of larger cohorts remains challenging. The Department of Dermatology and Allergy at the University Hospital of the Ludwig-Maximilians University (LMU) is one of the leading referral tertiary centers for cutaneous lymphomas in Germany, operating an interdisciplinary unit with dedicated consultations for affected patients. Consequently, this study represents a comparatively large cohort in the context of such a rare disease. Nevertheless, future prospective studies with larger sample sizes will be required to validate the present findings and further define the role of EVCM in routine dermatological practice. Another limitation concerns the varying levels of examiner experience in the use and interpretation of EVCM imaging. Since the images were available only in HE-mode and since the detailed visualization of specific cellular structures was limited, restricted expertise in recognizing CTCL features may have further complicated the diagnostic differentiation. Although examiners were guided by established EVCM features associated with CTCL (epidermotropism, Pautrier microabscesses, band-like infiltrates) and eczema (spongiosis, acanthosis, perivascular inflammation) during image assessment, the presence or absence of each individual feature was not systematically recorded for every case. The final classification therefore reflects an overall visual impression rather than a structured, feature-by-feature scoring approach. Consequently, the present dataset does not allow a retrospective analysis of which specific EVCM features were most strongly associated with correct CTCL recognition or with false-positive misclassification—an analysis that would be particularly informative for the seven cases that were repeatedly misclassified across examiners. We consider this an important direction for future research: a prospective study with structured, per-feature documentation for each lesion could identify the most diagnostically discriminative EVCM criteria and form the basis for a standardized, reproducible EVCM scoring system, potentially further improving diagnostic consistency across examiners of varying experience levels.

## 5. Conclusions

In conclusion, EVCM represents a promising technological adjunct that provides rapid, high-resolution assessment of freshly excised tissue and may facilitate earlier diagnostic orientation in CTCL, particularly mycosis fungoides, the predominant subtype in this cohort. The almost perfect interobserver agreement observed among examiners with differing levels of expertise further supports the reproducibility of EVCM-based assessment. EVCM should complement rather than replace conventional histopathological examination and may provide timely diagnostic support in the initial assessment of CTCL. Further studies are warranted to validate its diagnostic performance and to further define its role within diagnostic pathways for CTCL. Furthermore, incorporating artificial intelligence and machine learning algorithms into EVCM holds promise to improve diagnostic accuracy and streamline workflow, potentially enabling automated or semi-automated lesion assessment in the future.

## Figures and Tables

**Figure 1:**
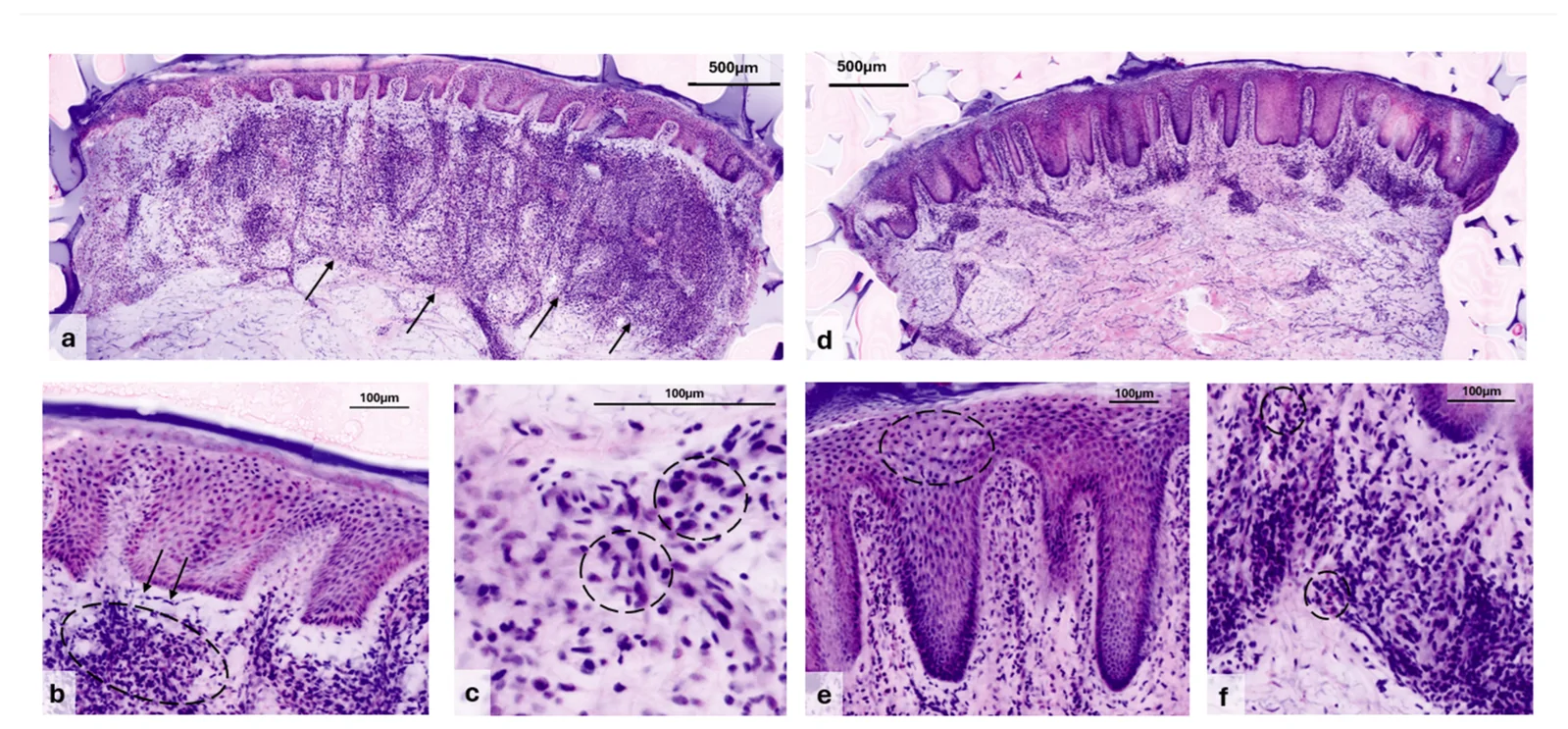
Ex vivo confocal microscopy of T-cell lymphoma (**a**–**c**). Ex vivo confocal microscopy of eczema (**d**–**f**). Ex vivo confocal microscopy of T-cell lymphoma in digital hematoxylin–eosin solution (**a**) showing a band-like papillary dermal lymphoid infiltrate (arrows). High-magnification view (**b**) with epidermotropic lymphocytes in a linear array (arrows) along the basal layer. A high-magnification view (**c**) showing a few atypical lymphocytes (circle), present in the underlying dermis, alongside smaller non-neoplastic lymphocytes. Ex vivo confocal microscopy of eczema in digital hematoxylin–eosin (**d**). A high-magnification view (**e**) showing the thickened epidermis (acanthosis) and intercellular edema (spongiosis) (dashed circle) in an eczematous lesion. Superficial dermis (**f**) with perivascular lymphocytic infiltrate, frequently mixed with eosinophils (circle).

**Figure 2:**
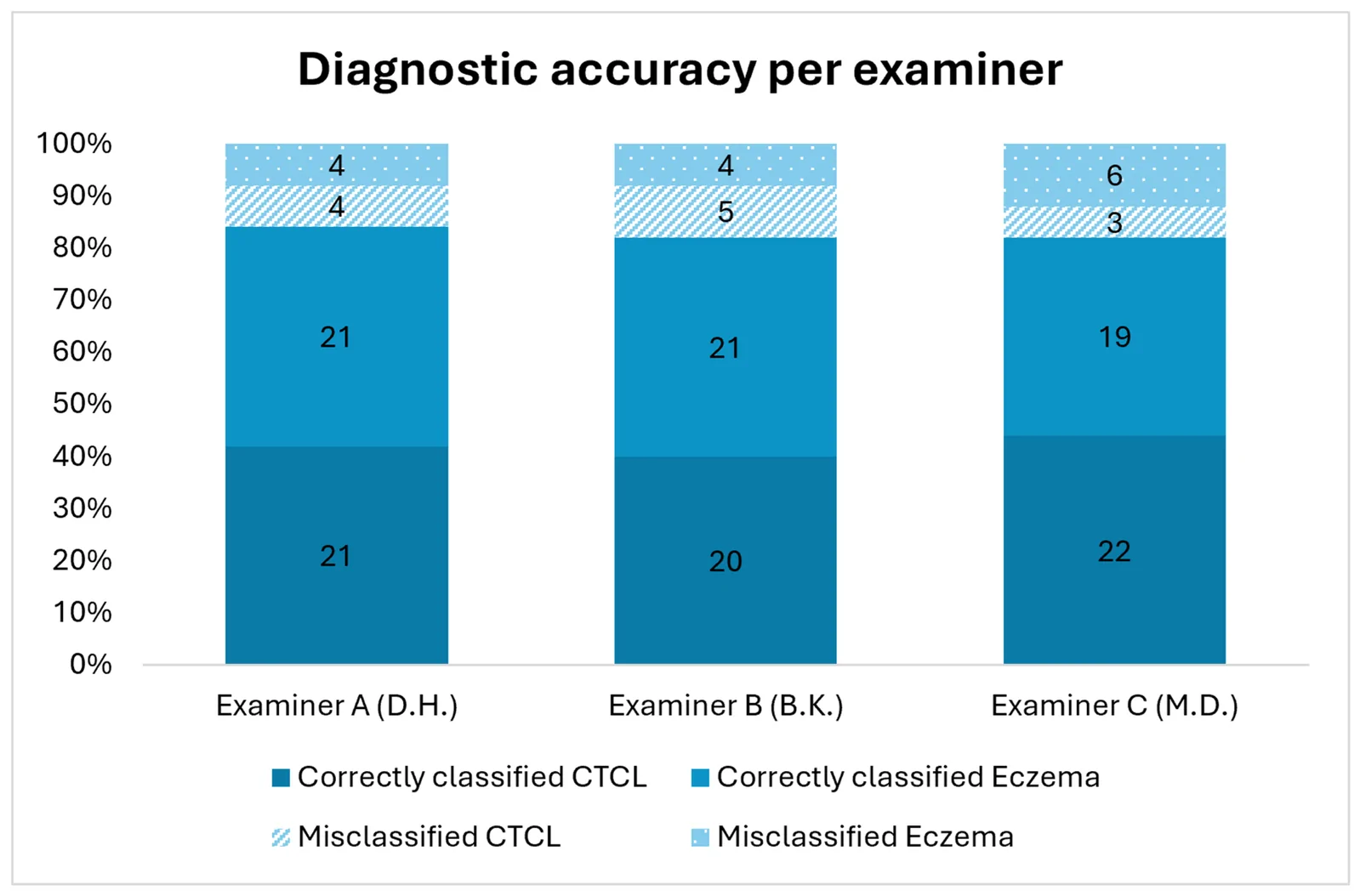
Diagnostic accuracy of lesion classification among the three investigators, Examiner A (EVCM-trained dermatohistopathologist), Examiner B (EVCM-unexperienced but dermatohistopathology-experienced), and Examiner C (EVCM-trained dermatologist without dermatohistopathology experience).

**Figure 3:**
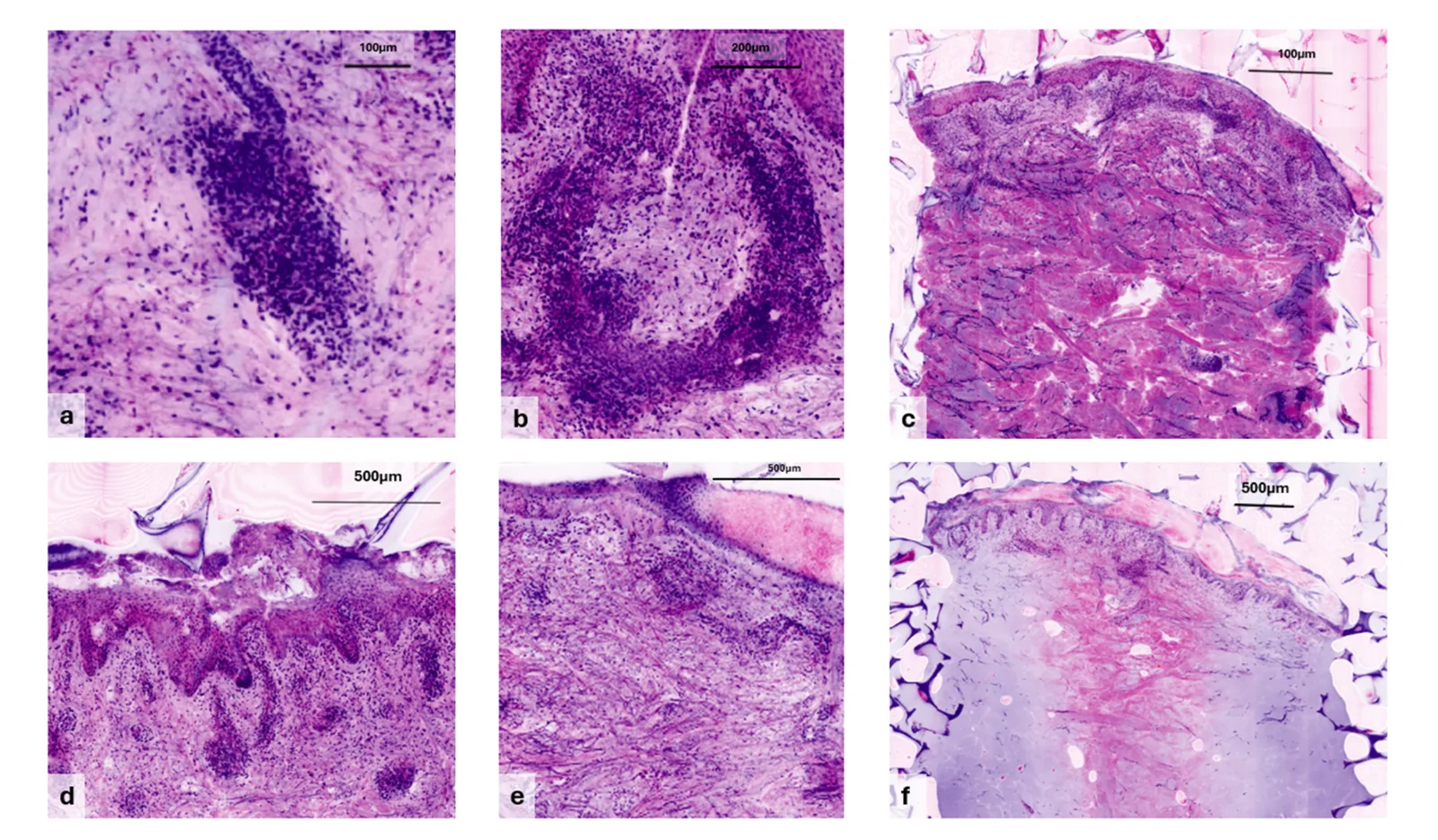
The major pitfalls. Ex vivo confocal images of eczema showing dense inflammatory infiltrate with overlapping inflammatory cells, complicating single-cell diagnostic analysis (**a**,**b**). Ex vivo confocal image of eczema shows excessively dark staining, impairing recognition and differentiation of individual cells and tissue structures (**c**). Ex vivo confocal images of eczema present epidermal damage and folding due to fixation, hindering structural and architectural analysis (**d**,**e**). Ex vivo confocal images of lymphoma show insufficient dye uptake, resulting in too light staining; tissue layers and individual cells are barely distinguishable, and the epidermis is strongly folded, limiting analysis (**f**).

**Table 1:** Diagnostic performance of each examiner, including sensitivity, specificity, positive predictive value (PPV), and negative predictive value (NPV) based on the classification of cutaneous T-cell lymphoma (CTCL) and eczema cases. Overall values and confidence intervals are based on the pooled classifications of all three examiners (Wilson score method) and do not account for within-lesion correlation between examiners, as the same 50 lesions were assessed by each examiner.

	Examiner A	Examiner B	Examiner C	Overall
Correctly classified CTCL	21	20	22	21
Misclassified CTCL	4	5	3	4
Correctly classified eczema	21	21	19	20.33
Misclassified eczema	4	4	6	4.67
Correctly classified TOTAL	0.84 (0.71–0.92)	0.82 (0.69–0.90)	0.82 (0.69–0.90)	0.83 (0.76–0.88)
Misclassified TOTAL	0.16 (0.08–0.29)	0.18 (0.10–0.31)	0.18 (0.10–0.31)	0.17 (0.12–0.24)
Sensitivity	0.84 (0.65–0.94)	0.80 (0.61–0.91)	0.88 (0.70–0.96)	0.84 (0.74–0.91)
Specificity	0.84 (0.65–0.94)	0.84 (0.65–0.94)	0.76 (0.57–0.89)	0.81 (0.71–0.89)
PPV	0.84 (0.65–0.94)	0.83 (0.64–0.93)	0.79 (0.60–0.90)	0.82 (0.72–0.89)
NPV	0.84 (0.65–0.94)	0.81 (0.62–0.91)	0.86 (0.67–0.95)	0.84 (0.73–0.90)

## Data Availability

The data underlying this study are available from the corresponding author upon reasonable request. Due to ethical and privacy considerations, the data are not publicly accessible.

## Abbreviations

The following abbreviations are used in this manuscript:

CBCL	Cutaneous B-cell lymphoma
CTCL	Cutaneous T-cell lymphoma
EVCM	Ex vivo confocal laser scanning microscopy
H&E	Hematoxylin and eosin
IHC	Immunohistochemistry
LMU	Ludwig-Maximilians University
LP	Lichen planus
MM	Malignant melanoma
NaCl	Sodium chloride
NPV	Negative predictive value
PBS	Phosphate-buffered saline
PPV	Positive predictive value
PTCL-NOS	Peripheral T-cell lymphoma, not otherwise specified
SCC	Squamous cell carcinoma
TNF α	Tumor necrosis factor α

## References

[1] Casey S., Aadil A. (2023). Dermatopathology, Cutaneous Lymphomas.

[2] Dobos G., Pohrt A., Ram-Wolff C., Lebbe C., Bouaziz J.D., Battistella M., Bagot M., de Masson A. (2020). Epidemiology of Cutaneous T-Cell Lymphomas: A Systematic Review and Meta-Analysis of 16,953 Patients. Cancers.

[3] Imam M.H., Shenoy P.J., Flowers C.R., Phillips A., Lechowicz M.J. (2013). Incidence and survival patterns of cutaneous T-cell lymphomas in the United States. Leuk. Lymphoma.

[4] Arulogun S.O., Prince H.M., Ng J., Lade S., Ryan G.F., Blewitt O., McCormack C. (2008). Long-term outcomes of patients with advanced-stage cutaneous T-cell lymphoma and large cell transformation. Blood.

[5] Scarisbrick J.J., Quaglino P., Prince H.M. (2019). The PROCLIPI international registry of early-stage mycosis fungoides identifies substantial diagnostic delay in most patients. Br. J. Dermatol..

[6] Klemke C.D., Mansmann U., Poenitz N., Dippel E., Goerdt S. (2005). Prognostic factors and prediction of prognosis by the CTCL Severity Index in mycosis fungoides and Sézary syndrome. Br. J. Dermatol..

[7] Martinez-Escala M.E., Posligua A.L., Wickless H., Rutherford A., Sable K.A., Rubio-Gonzalez B., Zhou X.A., Kaplan J.B., Pro B., Choi J. (2018). Progression of undiagnosed cutaneous lymphoma after anti-tumor necrosis factor-alpha therapy. J. Am. Acad. Dermatol..

[8] Hodak E., Amitay-Laish I. (2019). Mycosis fungoides: A great imitator. Clin. Dermatol..

[9] Bouwhuis S.A., Gonzalez-Arriaza H.L., McEvoy M.T., Davis M.D. (2003). Physical and mental health survey of 20 patients with Sézary syndrome using the medical outcomes study 36-item short-form health survey. J. Eur. Acad. Dermatol. Venereol..

[10] Demierre M.F., Tien A., Miller D. (2005). Health-related quality-of-life assessment in patients with cutaneous T-cell lymphoma. Arch. Dermatol..

[11] Dalal M., Mitchell S., McCloskey C., Zagadailov E., Gautam A. (2020). The clinical and humanistic burden of cutaneous T-cell lymphomas and response to conventional and novel therapies: Results of a systematic review. Expert Rev. Hematol..

[12] Demierre M.F., Gan S., Jones J., Miller D.R. (2006). Significant impact of cutaneous T-cell lymphoma on patients’ quality of life: Results of a 2005 National Cutaneous Lymphoma Foundation Survey. Cancer.

[13] Hartmann D., Krammer S., Bachmann M.R., Mathemeier L., Ruzicka T., Bagci I.S., von Braunmühl T. (2018). Ex vivo confocal microscopy features of cutaneous squamous cell carcinoma. J. Biophotonics.

[14] Vladimirova G., Ruini C., Kapp F., Kendziora B., Ergun E.Z., Bagci I.S., Krammer S., Jastaneyah J., Sattler E.C., Flaig M.J. (2022). Ex vivo confocal laser scanning microscopy: A diagnostic technique for easy real-time evaluation of benign and malignant skin tumours. J. Biophotonics..

[15] Deußing M., Buttgereit L., Maurer M., Swarlik A., Stärr L., Ohlmann A., Kerl-French K., Flaig M., Sattler E.C., French L.E. (2025). Benign or Malignant? Ex Vivo Confocal Laser Scanning Microscopy for Bedside Histological Assessment of Melanocytic Lesions. Cancers.

[16] Bağcı I.S., Aoki R., Krammer S., Vladimirova G., Ruzicka T., Sárdy M., French L.E., Hartmann D. (2020). Immunofluorescence and histopathological assessment using ex vivo confocal laser scanning microscopy in lichen planus. J. Biophotonics.

[17] Hartmann D., Stärr L., Maurer M., Stohldreier Y., Buttgereit L., Swarlik A., Sattler E.C., French L.E., Deußing M. (2025). Ex Vivo Confocal Laser Scanning Microscopy for Real-Time Pattern and Single-Cell Analysis in Inflammatory Skin Diseases. Exp. Dermatol..

[18] Razi S., Ouellette S., Khan S., Oh K.S., Truong T.M., Rao B.K. (2023). Role of VivaScope 2500 ex vivo confocal microscopy in skin pathology: Advantages, limitations, and future prospects. Skin Res. Technol..

[19] Smoller B.R., Santucci M., Wood G.S., Whittaker S.J. (2003). Histopathology and genetics of cutaneous T-cell lymphoma. Hematol. Oncol. Clin. N. Am..

[20] Newcombe R.G. (1998). Two-sided confidence intervals for the single proportion: Comparison of seven methods. Stat. Med..

[21] Fleiss J.L. (1971). Measuring nominal scale agreement among many raters. Psychol. Bull..

[22] Fleiss J.L., Levin B., Paik M.C. (2013). Statistical Methods for Rates and Proportions.

[23] Landis J.R., Koch G.G. (1977). The measurement of observer agreement for categorical data. Biometrics.

[24] Cochran W.G. (1950). The comparison of percentages in matched samples. Biometrika.

[25] McNemar Q. (1947). Note on the sampling error of the difference between correlated proportions or percentages. Psychometrika.

[26] Fisher R.A. (1922). On the interpretation of χ^2^ from contingency tables, and the calculation of P. J. R. Stat. Soc..

[27] Messner L., Deußing M., Maurer M., Buttgereit L., Stärr L., French L.E., Hartmann D. (2024). Ex Vivo Confocal Laser Scanning Microscopy in Rare Skin Diseases. Cancers.

[28] Assaf C., Dobos G., Zech I.M., Doess A., May M., Jadasz J. (2023). Epidemiologie reifzelliger T-und NK-Zell-Lymphome in Deutschland-Eine reprasentative Querschnittanalyse von GKV-Routinedaten: Epidemiology of mature T/NK-cell lymphomas in Germany-A representative cross-sectional study based on SHI claims data. J. Dtsch. Dermatol. Ges..

